# A Moment of Mindfulness: Computer-Mediated Mindfulness Practice Increases State Mindfulness

**DOI:** 10.1371/journal.pone.0153923

**Published:** 2016-04-22

**Authors:** Lynsey Mahmood, Tim Hopthrow, Georgina Randsley de Moura

**Affiliations:** Centre for the Study of Group Processes, School of Psychology, University of Kent, Canterbury, United Kingdom; Centre de Neuroscience Cognitive, FRANCE

## Abstract

Three studies investigated the use of a 5-minute, computer-mediated mindfulness practice in increasing levels of state mindfulness. In Study 1, 54 high school students completed the computer-mediated mindfulness practice in a lab setting and Toronto Mindfulness Scale (TMS) scores were measured before and after the practice. In Study 2 (*N* = 90) and Study 3 (*N* = 61), the mindfulness practice was tested with an entirely online sample to test the delivery of the 5-minute mindfulness practice via the internet. In Study 2 and 3, we found a significant increase in TMS scores in the mindful condition, but not in the control condition. These findings highlight the impact of a brief, mindfulness practice for single-session, computer-mediated use to increase mindfulness as a state.

## Introduction

Mindfulness intervention techniques traditionally have been delivered by a training program of several sessions, often requiring participants to invest a number of hours over the span of several weeks [1, 2]. A limitation to this method is that people may be unwilling or unable to invest this level of time, and indeed mindfulness may be beneficial in situations that arise spontaneously as part of everyday life (e.g., encountering a situation that may elicit stereotype threat, [3]). It would be impractical to expect, and unlikely to occur, that everyone who could benefit from mindfulness would engage in ongoing mindfulness practice. As such, it is important to determine whether a short mindfulness task can provide positive benefits for participants. Indeed, recent empirical research has shown positive effects of short 5-minute style mindfulness tasks on behavior and attitudes (e.g., [3–6]).

Previous research has largely focused on the efficacy of mindfulness courses and their impact on health outcomes (for reviews see: [7–13]). Evidence suggests that courses in mindfulness have a positive impact on outcomes for mental health, over long periods of time (such as stress, anxiety, depression, and aggression; [1, 2, 14–16]), but in some cases there is little or no evidence for positive effects (see [17]). Traditionally, mindfulness based stress reduction (MBSR) and mindfulness based cognitive therapy (MBCT) courses have been run over 8-weekly, one-hour, face-to-face sessions. They often include the use of tutorials and additional materials to guide meditators through practice, and are overseen by a professional practitioner [14, 18–20].

Although often beneficial, these prolonged sessions are not always feasible, and to combat this many courses are now accessible online, recognizing that it is important to make the positive potential benefits of mindfulness interventions accessible to a wider audience and to reduce costs [14, 15, 19–24]. Nonetheless, existing online courses still require a time commitment and some form of specialist input from therapists or practitioners. This type of practice may not be suitable for everyone, and the level of commitment required may not suit all situations. Our research takes a novel approach in that we are testing the effect of a 5-minute computer mediated mindfulness practice on state mindfulness.

Mindfulness is defined as enhanced attention and moment-by-moment awareness [25], a heightened state of involvement and wakefulness, being in the present [26], and maintenance of an open and non-judgmental consciousness. There are two views of mindfulness; one as a stable disposition or trait, which can be seen as an enduring aspect of personality and that can be maintained or enhanced through practice [27–29]. The other view is mindfulness as a skill or *state*. State mindfulness is viewed as purposeful attention. That is, only whilst the individual purposefully brings their attention to the practice of mindfulness, are they able to step outside of automated perceptual processing and focus their attention on minute details of mental activity that would not be noticed usually [7]. In other words, a mindful *state* is only maintained while attention is intentionally cultivated, and when attention is no longer regulated in this way, the mindful state will cease [7]. Although separate constructs, it is likely that individuals will have a stable level of trait mindfulness and altering levels of state mindfulness (e.g. as is for anxiety, anger etc., [30]).

The TMS [31] is based on Bishop et al.’s [7] two-component definition of state mindfulness, comprising of self-regulation of attention and orientation to experience. The TMS is a measure of *an individual’s level of mindfulness at a single point in time* (i.e. the current mindful state) rather than as a stable individual difference measure or as the ability to evoke a mindful state [31]. Our research tests whether a short 5-minute mindfulness practice is sufficient to increase levels of state mindfulness using the TMS measure, which assesses curiosity and decentering [31].

Single session mindfulness practice has been applied outside of clinical settings, and has been shown to reduce the negative effects of stereotype threat on women’s mathematics performance [3], reduce aggressive responses to social threat [4], and reduce the likelihood of committing the correspondence bias when judging other people’s behavior [5]. This suggests that mindfulness practice could be beneficial in social settings and have applications beyond clinical and health psychology. With the proliferation of accessing mindfulness practice online (including via smartphones), it is important to understand whether brief mindfulness practice increases levels of state mindfulness, and thus whether such salutary effects are the result of mindfulness itself.

Johnson et al. [32] highlighted the importance of disentangling the effects of one-session mindfulness from those of multiple sessions of mindfulness. They outlined that brief mindfulness formats, including three to five sessions of mindfulness meditation, can have beneficial effects [33–36], but that mindfulness has only been measured once all of the mindfulness sessions have been completed [32]. In addition, studies that have used only one session of brief (< 30 minutes) mindfulness practice either measure mindfulness at the end of the study [3, 5], did not measure state mindfulness at all [33, 37, 38] or supplemented practice with further information about mindfulness practice and the positive outcomes it can elicit [39]. Methodologically, this means that there is no pre-practice baseline marker with which to compare any improvements or changes in mindfulness. It is also not possible to attribute any changes in outcome behaviors, or measured mindfulness levels, to the practice itself. There is the potential here that these positive outcomes are artifacts of the information participants have learned about the benefits of mindfulness, or a result of demand characteristics, rather than the practice itself.

In order to better understand whether changes in behavioral outcomes are likely to be the result of mindfulness, research is required to test whether state mindfulness is higher after a brief mindfulness practice delivered via computer software, in a short time period, without additional information or support. We present three studies testing the effect on state mindfulness of a 5-minute mindfulness practice versus a control, in a laboratory environment (Study 1), and via online software (Studies 2 & 3). To address the limitations of previous research as detailed above, participants are not given any information about mindfulness practice or its effects. State mindfulness is measured before and immediately after practice to show any changes in levels of state mindfulness.

It is expected that those who completed the mindfulness exercise will report a greater increase in scores on the TMS compared to those in the control condition, suggesting an increase in state mindfulness after a 5-minute practice.

### Ethics statement

The study was approved by the Psychology Ethics Committee at the University of Kent and is in line with British Psychological Society ethical guidelines for participants aged over 16 years. Participants were briefed verbally before the study, including what consent means. The participants themselves gave consent within the survey software. Verbal consent was received from the participants’ teachers from the high school, who also remained present throughout the study, but were not directly involved in data collection. For Study 2 and 3, all participants provided their informed consent by clicking in agreement within the online survey software. This means of obtaining consent was approved by the Psychology Ethics Committee at the University of Kent.

## Study 1

### Method

#### Materials

Mindfulness Practice: The mindfulness audio file consisted of a 5-minute mindfulness body scan, in which participants were asked to use their breath as an anchor to help focus on the present moment (adapted from [40]). Participants were guided through focusing on the sensations in their body sequentially from foot to head. For example, “shifting attention up from there now into the torso, being aware of the back region, the chest, the abdomen”. Similar body scan mindfulness techniques have been used in previous research as part of a six to eight week mindfulness course [18–20, 24, 41] and in one off laboratory sessions[42–44]. The body scan practices used in previous research have typically ranged in length from 10 to 45 minutes.

Here, a 5-minute version was developed for two reasons. First, we were interested in whether as little as 5-minutes of mindfulness practice has any effect on levels of state mindfulness. Second, we were interested in developing a practice that could be applied as practically as possible to everyday settings such as the classroom or workplace, where pausing to practice mindfulness for longer periods may not be feasible. The audio was purposefully developed *excluding* any mention of mindfulness. This was to try and avoid any demand characteristics in participants who may have some knowledge of the beneficial effects of mindfulness practice.

In the control condition, participants were asked to take a few deep breaths and await further instructions, there was then a 4-minute silence before these instructions were repeated and participants were able to continue the questionnaire. This control condition was chosen since it allowed us to control the length of the audio files that participants were listening to, and keep the timing as similar as possible for all participants. Although Wilson et al. [45] suggest that individuals do not like to be left with their own thoughts, even for short periods of time, the authors do also point out that those who were left with nothing to do reported a far greater amount of mind-wandering, which may also be inversely related to mindfulness [46]. In addition, Hopthrow et al. [5] compared a 5-minute mindfulness practice to the same type of control condition *and* to an attention to detail task, and found that the mindfulness condition significantly reduced the extent that participants committed the correspondence bias, but that the control and attention conditions did not differ significantly. This demonstrates that state mindfulness is not the same as attention to detail. Other research has utilized listening to audio book excerpts as a control condition [32, 42], but these are for longer periods of time than 5-minutes. We were also particularly interested in practical applications of the brief mindfulness practice, and so attempted to use a control condition that would be comparable to individuals’ daily experience- for example, being at work and losing focus on the present task for a short period may involve doing nothing, but not necessarily listening to an audio book.

To ensure that all participants experienced as similar conditions as possible, the questionnaire software was programmed so that the audio files played for the full five minutes and participants were not able to move away from this page until the audio was finished. In addition, the audio files for both the mindfulness and control conditions in all studies were recorded using the same male voice to ensure consistency.

State Mindfulness Measure: The TMS scale [31] was presented before and after the mindfulness (vs. control) exercise. All items were randomized to try and reduce the likelihood that participants recognized the questionnaire and responded based on their previous answers. All items were measured on a 5-point scale (*1 = not at all*, *5 = very much*), with higher scores indicating higher levels of state mindfulness.

#### Participants and Design

Fifty-four students (51 females, two males, and one undisclosed, *M*_*age*_ = 17, ranging from 16 to 18 years) from a local high school, attending an introductory psychology visit day at the University of Kent, took part in the study. Participation was voluntary, and no incentives were given. The TMS was measured before and immediately after the mindfulness (vs. control) exercise. Participants were allocated randomly to either the mindfulness (*N* = 27) or control (*N* = 27) conditions, allocation was double blind so neither the participant nor the experimenters were aware which condition any participant was in.

#### Procedure

Participants were gathered in large computer room and each seated at a computer station with headphones. Participants, were seated next to one another with no dividers between the computer stations. Participants were told that they would be asked to listen to audio files that might contain some pauses of varying lengths, but that the survey software was programmed to move to the next page when the audio had finished, so participants would be required to keep their headphones on for the duration of the study. This also ensured that participants were unaware of the length of audio, and both participants and researchers were blind as to who was in which condition.

A brief introduction to the session was given by the researchers, outlining what the participants could expect in the study and relevant ethical considerations. Once logged into the survey software, participants first received a written information sheet and were asked to indicate their consent. The TMS was then presented, followed by either the mindfulness or control audio file. After the 5-minute audio, participants were presented with the TMS again. Participants were then given a written debrief and thanked before having the opportunity to ask the researchers any questions about the study or methodology.

### Results and Discussion

Descriptive statistics for the TMS at time 1 and time 2 are presented in Table 1. A 2 (Condition: mindfulness vs. control) x 2 (Time: time 1, time 2) mixed ANOVA was run with Time entered as within-participants. There were 27 participants in each condition.

**Table 1:** T1 and T2 Mean (Standard Deviation) scores for the TMS.

	T1	T2
	TMS	TMS
Mindful	2.67 (0.69)	2.87 (0.66)
Control	2.73 (0.56)	2.67 (0.78)

There were no significant main effects of Condition (*F* (1, 52) = 0.18, *p* = .68, η^2^ < .01), or Time (*F* (1, 52) = 0.60, *p* = .44, η^2^ = .01). The interaction of Condition x Time was non-significant, *F* (1, 52) = 2.17, *p* = .15, η^2^ = .04.

Although differences were expected, there were some limitations in Study 1 which may have impacted the results. Firstly, the full TMS scale was completed by participants before and immediately after the mindfulness (vs. control) audio files. Therefore, it is possible that participants remembered questions and responses at T2 and answered in line with their T1 responses. In addition, the sample comprised of students seated in an open-plan space where there was the opportunity to distract each other, or for enhanced evaluation apprehension where peers could see whether participants had followed instructions, for example to keep their eyes closed. The results may have been weakened by extraneous methodological factors.

Study 2 addresses these issues by allowing participants to complete the survey in their own choice of surroundings, and by separating the TMS into two subscales and counterbalancing the order in which they were completed.

## Study 2

### Method

#### Participants and Design

Ninety participants recruited from Amazon’s Mechanical Turk (MTurk), who were residents of the U.S.A, took part in the study in return for a small monetary payment. This is a suitable recruitment platform as it provides a wider age range than student samples [47]. The survey software allocated participants randomly to either a mindfulness (*N* = 51) or control condition (*N* = 39) and also randomly to complete either the decentering TMS subscale first, followed by the curiosity TMS subscale (*N* = 35), or the curiosity subscale first, followed by the decentering subscale (*N* = 55).

#### Materials and Procedure

Study 2 used the same materials as in Study 1, and the procedure differed in only two ways. First, Study 2 was delivered entirely online, meaning that participants were able to log in and complete the survey at any time and in any location with internet access. Second, the TMS subscales were separated and one was presented before the audio file, and the other after (presentation order was counterbalanced), meaning that participants only saw each subscale of the TMS at each time point. This was done to ensure that the questions in the TMS subscales were not in themselves weakening the effects of the intervention. Separating the TMS subscales provided a mechanism to reduce the chances that the wording of the questions was influencing state mindfulness.

### Results and Discussion

A 2 (Condition: mindfulness vs. control) x 2 (Presentation Order: decentered pre-audio vs. curiosity pre-audio) x 2 (TMS subscale: decentering vs. curiosity) mixed ANOVA was conducted, with TMS subscale as a within-participants factor.

There was no main effect of Presentation Order, *F* (1, 86) = 0.37, *p* = .54, η^2^ < .01. There was no main effect of TMS subscale, *F* (1, 86) = 3.03, *p* = .09 η^2^ = .03. There was a significant main effect of Condition, *F* (1,86) = 9.85, *p* < .01, η^2^ = .10, whereby overall TMS scores from the mindful condition (*M* = 3.17) were significantly higher than those in the control condition (*M* = 2.61). None of the two way interactions were significant, condition x presentation order: *F* (1, 86) = 0.28, *p* = .60, η^2^ < .01; TMS subscale x condition: *F* (1, 86) = 0.88, *p* = .35, η^2^ < .01; TMS subscale x presentation order, *F* (1, 86) = 2.42, *p* = .12, η^2^ = .03. There was a significant three way interaction of Condition x Presentation Order x TMS subscale, *F*(1,86) = 4.49, *p* < .05, η^2^ = .05.

#### Simple Effects Analysis

**Fig 1:**
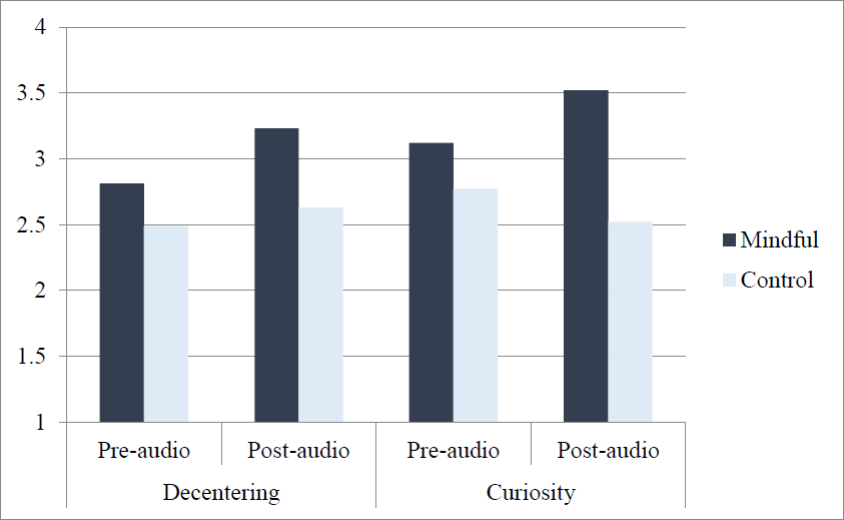
The effect of mindfulness condition on TMS subscales as a function of presentation order.

Participants who completed the decentering subscale first, scored significantly higher on the curiosity subscale post-audio in the mindful condition (*M* = 3.52, *SD* = 0.86) than the control condition (*M* = 2.52, *SD* = 1.12), *F* (1, 86) = 8.21, *p* < .01, η^2^ = .09. This was also true for those who completed the curiosity subscale first, although the effect was slightly weaker, with scores on the decentering subscale post-audio were significantly higher in the mindful condition (*M* = 3.23, *SD* = 0.74) than in the control condition (*M* = 2.63, *SD* = 0.79), *F* (1, 86) = 6.69, *p* < .05, η^2^ = .07. Table 2 and Fig 1 show that the mindfulness condition did increase levels of state mindfulness compared to the control.

**Table 2:** T1 and T2 Mean (Standard Deviations) scores for TMS.

	Decentered T1	Curious T1	Decentered T2	Curious T2
Mindful	2.81 (1.12)	3.12 (1.20)	3.23 (0.74)	3.52 (0.86)
Control	2.49 (0.75)	2.79 (1.01)	2.63 (0.79)	2.52 (1.12)

There were no significant differences across either the mindfulness or control conditions in mean scores of decentering and curiosity between the groups who completed the decentering subscale first and those who completed the curiosity subscales first (all *p*s > .09). This shows that there were no significant differences in mean levels of the two subscales between participants at pre-or post-audio, and across both mindfulness and control conditions. This suggests that the results from Study 1 were not simply the result of memorized responses.

Taken together, this supports the hypothesis that state mindfulness would be significantly increased after the brief mindfulness practice, but would not after no practice, and suggests that delivery of the mindfulness practice via the internet would be feasible. The finding that the two subscales did not differ between participants suggests that when combined, the online sample showed an increase in both curiosity and decentering. However, the separation of the two TMS subscales means that it is not possible to see the differences in scores between pre- and post-intervention in the online sample. With this in mind, Study 3 extends these findings by asking participants to complete the full TMS scale before, and immediately after, the condition but unlike Study 1 using the online methodology.

## Study 3

### Method

#### Participants and Design

Sixty-one participants (37 male and 24 female, *M*_*age*_ = 33.56, ranging from 18 to 70 years) were recruited via MTurk. Participants were residents of the U.S.A and received a small monetary incentive for participation. The study was a 2 (Condition: mindfulness vs. control) x 2 (Time: 1, 2) mixed factor design, with Time as a within-participants factor. There were 28 participants in the control condition and 27 in the mindfulness condition.

#### Materials and Procedure

The same materials were used as in Study 1 and Study 2. The whole TMS was presented to participants before and immediately after the audio (as in Study 1) and the survey was delivered entirely online (as in Study 2).

### Results and Discussion

Descriptive statistics for the TMS at time 1 and time 2 are presented in Table 3. Data was analysed with a 2 (Condition: mindfulness vs. control) x 2 (Time: time 1, time 2) mixed ANOVA, with the Time as a within-participants factor.

**Table 3:** T1 and T2 Mean (Standard Deviation) scores for state mindfulness (TMS).

	T1	T2
	TMS	TMS
Mindful	3.17 (0.91)	3.49 (0.82)
Control	2.81 (0.86)	2.71 (0.96)

There was a significant main effect of Condition, *F* (1, 59) = 7.33, *p* < .01, η^2^ = .11, showing that those in the mindful condition scored significantly higher on state mindfulness (*M* = 3.33, *SD* = 0.82) than those in the control condition (*M* = 2.76, *SD* = 0.96). The main effect of Time was non-significant, *F* (1, 59) = 1.78, *p* = .19, η^2^ = .03. The interaction of Condition x Time was significant, *F* (1, 59) = 5.56, *p* < .05, η^2^ = .09.

#### Simple Effects Analysis

The simple effects of the interaction between condition and time show that, in the control condition, there were no significant differences in the state mindfulness scores at T1 compared to T2, *F* (1, 59) = 0.55, *p* = .45, η^2^ = .01. As expected, for participants in the mindfulness condition, the reported state mindfulness was significantly higher at T2 than at T1, *F* (1, 59) = 6.49, *p* < .05, η^2^ = .10.

Importantly, there were no significant differences in TMS scores at T1 between the mindfulness and control conditions, *F* (1, 59) = 2.50, *p* = .12, η^2^ = .04. The scores at T2 did differ between the mindfulness and control conditions, *F* (1, 59) = 11.51, *p* < .01, η^2^ = .16. This showed that at T2 those in the mindful condition scored significantly higher on state mindfulness as measured by TMS (*M* = 3.49) than those in the control condition (*M* = 2.71).

The results from Study 3 extend those of Study 1 and Study 2, showing that as little as five minutes of computer-mediated mindfulness practice elicits an increase in state mindfulness.

## General Discussion

The current research suggests that 5-minutes of mindfulness practice is enough to elicit increases in state mindfulness, when delivered online. To our knowledge this is the first study to empirically test the use of a 5-minute mindfulness exercise in terms of changes to state mindfulness levels pre- and post-practice, and to investigate this in the context of delivering the practice online, with no other information or specialist input. A 5-minute mindfulness task has been used in previous research in the laboratory [3–5], however this utilised a mindful raisin eating practice. Since the purpose of this research was to empirically assess the use of computer-mediated practice, it was not possible to use the mindful raisin eating practice, and so a body scan was adapted for use as a 5-minute practice.

Previous research has shown that brief mindfulness practices have been used without measuring levels of mindfulness [6, 33, 37, 38] or with the use of additional materials [39], thus assuming that practice leads to increased mindfulness. However, none have looked at changes to levels of mindfulness after the use of a brief practice and whether as little as 5-minutes would be enough to elicit these changes.

Study 1 did not show an impact of the brief mindfulness intervention in the laboratory setting, although the trend was in the right direction. The findings from Studies 2 and 3 suggest that this is likely to be due to the experimental conditions, the nature of the mindfulness condition being delivered in a group laboratory setting.

When the 5-minute mindfulness practice was delivered via the internet, Study 2 showed that there were differences in state mindfulness between a mindfulness and control condition. On refining the paradigm, Study 3 showed that computer-mediated mindfulness practice elicited an increase in TMS scores. This provides evidence that the use of a brief mindfulness practice with a non-clinical sample, and without any specialist input is effective in increasing levels of state mindfulness. Although a number of mindfulness practices exist that are readily available to the general population through smartphone apps and websites, this is the first study to examine whether such practices are effectively increasing state mindfulness.

The findings from the present research suggests that allowing participants to carry out interventions in their own surroundings, with greater anonymity, may be the cause of increases in the effectiveness of interventions [48, 49] and that this is true even when the practice is very brief, and the participants are not using the practice to alleviate clinical sympotms. Taken together the studies presented in this research show that 5-minutes of mindfulness practcie effectively increase levels of state mindfulness, and that delivering practice online so that participants can practice in their own time/surroundings is effective.

Further behavioral measures were not included in the present research since previous research has suggested brief mindfulness practice has beneficial effects in social domains such as stereotype threat, social rejection, and judging others behavior [3–5]. However, this is the first empirical investigation of whether mindfulness practice itself is increasing state mindfulness, something that previous research has assumed. However, the present findings suggest that 5-minute mindfulness practices, delivered online could be applied to different research questions, and practical contexts, and also have a positive impact on the number of individuals who can access mindfulness practice, without the requirements for costly expert training and reliance on individuals’ motivation to commit to long courses.

A future direction for research is to consider how long the mindful state lasts. The studies presented show that state mindfulness is enhanced after practice, but only that this effect is immediate and it is not yet know how long this effect lasts for. It would be important to compare different types of mindfulness practice as well as whether a longer mindfulness practice leads to a longer state of mindfulness.

The studies presented are not without limitations. Participants in the online samples were not asked about their chosen surroundings and were assumed to be alone at the time of practicing the mindfulness exercise. In the context for which it is thought that a breif, computer-mediated mindfulness practice would be beneficial (such as organisations or classrooms), background noise and some slight disctrations in the environment are likely to be unaviodable, and may in fact increase the strength of these findings. However, future studies could ask participants the extent to which they were focused on the task or perhaps use mouse tracking to see whether participants are clicking elsewhere, perhaps viewing other webpages during the audio. In particualr this could shed light on what participants are doing in the control condition, where they are left in silence for the duration of the 5-minute audio file. Alternative control conditions may also provide greater insight into the process by which mindfulness is having an effect. For example, Hopthrow et al. [5] have shown that mindfulness differs to an attention to detail task, but that attention to detail did not differ from the passive control condition.

The Mindfulness Attitudes Scale [50] has also been used to control for participants openness to mindfulness practice, which indicates the level to which participants were willing to engage in the practice. However this relies on participants understanding what mindfulness is, which may be particualrly varied depending on the cotext in which mindfulness is applied [51]. In addition, the present research relied on self-reported levels of state mindfulness, which may also have been influenced by participants’ level of understanding or contextual knowledge of mindfulness and meditation. Although Study 2 separated the TMS subscales to ensure participants levels of state mindfulness were not being impacted by memory of questionnaire items, future research should consider more innovative ways to measure mindfulness and also consider previous mindfulness experience.

Age is another factor to consider since Cavanagh et al. [52] point out that the privacy and anonymity of online practice is particulalry appealing to younger individuals. This is pertinent to the sample in Study 1, since anonymity was reduced by the fact that although the practice was individual, they were still sat in a large open room amongst peers. Participants in Study 2 and Study 3 were generally older than those in Study 1, so future research could consider the use of the online mindfulness practice with a younger sample. However, age was not a key factor in the current research and despite the possible limitations of the younger sample, the findings support the notion that a 5-minute, computer-mediated mindfulness exercise, with no practitioner input increased participants’ state mindfulness.

In conclusion, the current research addresses an important gap in the current literature on mindfulness. That is, empirically measuring changes to state mindfulness and testing the effectiveness of a brief mindfulness practice. The studies presented show that as little as 5-minutes of mindfulness is enough to elicit increased state mindfulness. In addition, in the context of computer-mediated practice, the 5-minute mindfulness practice can be delivered effectively with no specialist input, and is effective when delivered online where the participant is able to choose their own surroundings to carry out the practice. This has implications for being able to apply mindfulness into individuals’ daily lives. A 5-minute practice can be used alone to increase state mindfulness, without the additional time and resources that mindfulness courses require. The next step is to investigate whether this brief practice has positive behavioural outcomes, in the same way that mindfulness courses can have.
